# Experiences of Patient-Centered Care Among Older Community-Dwelling Australians

**DOI:** 10.3389/fpubh.2022.912137

**Published:** 2022-06-14

**Authors:** Breanne Hobden, Elise Mansfield, Megan Freund, Matthew Clapham, Rob Sanson-Fisher

**Affiliations:** ^1^Health Behaviour Research Collaborative, School of Medicine and Public Health, Faculty of Health and Medicine, University of Newcastle, Callaghan, NSW, Australia; ^2^Priority Research Centre for Health Behaviour, University of Newcastle, Callaghan, NSW, Australia; ^3^Hunter Medical Research Institute, New Lambton Heights, NSW, Australia

**Keywords:** patient care, patient-centered care, community-dwelling, older adults, aged care, perceptions, healthcare, consultation

## Abstract

**Background:**

Older adults represent the largest consumers of health care. It is, therefore, important that they receive adequate patient-centered care to empower them to be proactive in managing their health.

**Aims:**

This study examined the proportion of older community-dwelling individuals who report receiving patient-centered care during healthcare consultations.

**Methods:**

A cross-sectional study was conducted with 117 clients of an Australian aged care provider. Clients completed a survey examining their perceptions of whether they received patient-centered care (11-items) from healthcare professionals.

**Results:**

The mean number of patient-centered care items reported was 8.7 (±3.1). Speaking to the patient with respect was the item most often reported to be patient-centered (94%). Asking patients about treatment goals or expectations (62%) and how involved they would like to be in treatment (67%) were the items least reported to be patient-centered.

**Conclusion:**

Older adults perceived some important aspects of care were not provided with a patient-centered approach. There is a need to improve healthcare providers' elicitation of older patients' care preferences, enabling patients to determine their level of involvement in their health management.

## Introduction

Older adults represent the largest consumers of health care (1, 2). This is due to increased rates of chronic conditions among older adults, including higher rates of multi-morbidity (1). Managing one or more chronic conditions, as well as the physical effects of aging (e.g., frailty), often result in complex health care management for older adults. As the aging populations continues to grow, there is a need to ensure that the healthcare needs of older people are met and that they feel confident in their ability to manage their health.

Patient-centered care is widely accepted as a pillar of high quality medical care (3). Patient centered care places responsiveness to the patients' needs, values and preferences at the forefront of medical care (3). It has been associated with increased medication adherence (4), decreased healthcare utilization (5), and improved health outcomes (6, 7). The importance of patient-centered care for vulnerable population groups, such as the older population and people with dementia (8), patients with multi-morbidity (6) and chronic heart failure (7) has been highlighted in the literature.

While patient-centered care is considered a key component of high-quality health care (3), the move from a paternalistic healthcare model to a more patient-centered shared care approach continues to be difficult to implement in to practice (9). The paternalistic model of healthcare positions the provider as the expert in patient care and therefore the key decision-maker (10). This model has been the traditional approach up until the last few decades and does not enable the patients' individual needs or preferences to be accounted for in the decision-making process. Studies have consistently demonstrated gaps in patient-centered care delivery across a range of chronic disease groups (11–13) and health care settings (9, 14, 15). For instance, a study examining receipt of patient-centered follow-up care for 239 patients who completed cancer treatment demonstrated only 49% received all of the examined care aspects (13). Another study conducted among more than 1,400 general practice patients demonstrated higher rates of patient-centered care, with 83% reporting receipt of all the examined care aspects (15). While these studies demonstrate variation in self-reported receipt of patient-centered care, they highlight a need for improvement in this area.

Internationally, policies and initiatives are being developed to help older adults remain community-dwelling for as long as possible (16). High quality healthcare, which includes a patient-centered approach, is an integral part to ensuring older adults are able to manage their health at home. Nevertheless, there are several challenges to implementing patient-centered care for older community-dwelling adults. Negative attitudes, ageist stereotypes, prejudice and discrimination toward older adults in the healthcare system have been reported (2). These views may, in turn, impact healthcare providers' preconceptions about older adults' desires and abilities to be involved in decision making for their care. There may also be difficulties in engaging older adults in person-centered care processes, particularly for those with cognitive impairment (17), hearing loss and communication difficulties (18). Limited research has explored patient-centered care among community-dwelling older adults (19–21). To the authors' knowledge, no quantitative descriptive studies have been conducted to explore community-dwelling older adults' perceptions of patient-centered care during interactions with a healthcare professional. Increased understanding of patient-centered care can better inform policies and initiatives to support older adults to remain community-dwelling.

## Aims

To examine the proportion of older community-dwelling individuals who report receiving patient-centered care during consultations with healthcare professionals.

## Methods

### Design and Setting

This cross-sectional descriptive survey study was conducted with clients receiving care from one not-for-profit, Australian government-funded aged care provider. In Australia, community-based aged care providers deliver services such as personal care, domestic assistance and support with medications. The participating aged care provider delivers services to over 8,000 older Australians living in the community in rural, regional, and remote areas. Participants for this study were recruited from three Australian states. Ethics approval was granted by the University of Newcastle Human Research Ethics Committee. Participants provided written informed consent.

### Sample

Eligible clients were those who were: receiving home care services from the participating aged care provider; considered by their Case Manager to be physically and cognitively capable of providing informed consent; and able to complete an English language survey. Clients who were too ill to complete the survey, overseas, were recently bereaved, or were on a waiting list for a permanent place in a residential aged care facility were ineligible.

### Participant Recruitment

Case Managers (*n* = 30) working with the Aged Care Provider performed recruitment and data collection for the study. The Case Managers role involves providing assessments, developing care plans, managing budgets and liaising with health professionals on behalf of their clients. Case Managers were identified *via* staff lists and invited *via* email to participate. They participated in a 1-h training session that detailed the study and the survey's administration that was led by a member of the research team (BH). All invited Case Managers consented to undertaking the study. Potentially eligible clients of participating Case Managers were identified by a staff member at the aged care provider. Case Managers further reviewed the client list to ensure the included patients were considered cognitively able and were not on a waiting list for an aged care facility. Of the remaining sample, a random computer generator was used to select 400 clients to participate in the study. Identified clients were mailed a recruitment package by the aged care provider, including an information statement and consent form. Case Managers followed up with the identified clients either by telephone or at the next scheduled appointment to confirm the client's eligibility and obtain informed consent.

### Data Collection

During a scheduled home visit, Case Managers administered a web-based survey with consenting clients *via* a computer tablet, with pen-and-paper surveys available in case of technical difficulties. To reduce participant burden, the survey questions were administered across two sessions conducted 3 months apart.

### Measures

The data reported in this study were collected as part of a larger research study. Only measures pertaining to the current research question are provided here.

#### Demographic Variables

In survey 1, patients self-reported their age, gender, highest level of education, Aboriginal or Torres Strait Islander status, marital status, living arrangements, private health insurance status, and home postcode.

#### Previous Experience With Healthcare Professionals

In survey 2, participants were asked to self-report their experiences of receiving patient-centered care in general during appointments with healthcare professionals (seven items), and when discussing possible treatments with their healthcare professionals (four items). The items were developed by the research team, following a review of the literature on principles of patient-centered care for older adults (3, 18, 22) and refined in consultation with consumers. Participants were asked to respond using a four-point response scale (“Yes, and I wanted this;” “Yes, but I didn't want this;” “No, but I wanted this;” “No, but I didn't want this”). This response scale allowed for an examination of whether or not care received was consistent with clients' preferences. The reading age for the survey was under an 8th grade level according the Flesch Kincaid Reading Ease test and items were piloted with five participants prior to data collection.

## Analysis

Counts and percentages of non-missing observations for categorical variables and mean with standard deviation (SD) for continuous variables were calculated. A “received patient-centered care” variable was created and was defined as receiving care consistent with preferences (i.e., Yes, and I wanted this or No, but I didn't want this). A “did not receive patient-centered care” variable was created and was defined as receiving care inconsistent with preferences (i.e., Yes, but I didn't want this or No, but I wanted this). The number of “received patient-centered” items was calculated for each participant, with a maximum score of 11. Postcode was used to categorize remoteness using the Accessibility/Remoteness Index of Australia (ARIA). Statistical analyses were undertaken using R version 4.0.3 (2020-10-10; R Foundation for Statistical Computing, Vienna, Austria).

## Results

Of the 400 randomly selected clients, 357 were approached to participate in the study and 295 were eligible. Consent was provided by 158 participants (54% consent rate) and 117 participants were retained at the 3-month follow-up (74% retention rate) and were included in the current analysis. Sixteen participants had missing demographic data (14%). Most participants were female (65.3%), with an average age of 78 years (SD = ±8.5) and had a high school education or below (72.3%; see Table 1 for full characteristics).

**Table 1:** Participant demographics (*n* = 101).

**Characteristic**	**Categories**	***N* (%)**
Age	Mean (SD)	78.0 (8.5)
Gender	Male	34 (33.7)
	Female	66 (65.3)
	Other	1 (1.0)
Education	High School or below	73 (72.3)
	Trade or vocational education	21 (20.8)
	University or postgraduate degree	7 (6.9)
Aboriginal or Torres Strait Islander	Yes, Aboriginal	4 (4.0)
Marital status	Married or living with partner	33 (32.7)
	Divorced or separated	15 (14.9)
	Widowed	43 (42.6)
	Never married	10 (9.9)
Living arrangements	Lives alone	59 (58.4)
Private health insurance	Yes	37 (36.6)
Remoteness	Inner/Outer Regional	73 (74.5)
	Major Cities	25 (25.5)

Table 2 presents the participant responses for the 11 patient-centered care items. Speaking to the patient with respect was the aspect of care most commonly reported to be patient-centered (94%, *n* = 108). Items with the lowest proportion of participants indicating that they received patient-centered care included whether health care professionals generally: asked the patient about goals or expectations of treatment (61.7%, *n* = 71); asked how involved the patient would like to be in treatment (67%, *n* = 77); encouraged the patient to ask questions (69%, *n* = 80); and helped to weigh up the pros and cons of different treatment options (75%, *n* = 87). The majority of these responses consisted of patients reporting, “No, but I wanted this” (21–37%). The total patient-centered care score was calculated for 106 participants who answered all 11 items of care. Of these participants, 42% (*n* = 44) received all 11 care aspects in agreement with their wishes, while 3.8% (*n* = 4) did not receive any of the care items in agreement with their wishes. The mean number of items for which participants perceived receiving patient-centered care was 8.7 (±3.1) out of 11.

**Table 2:** Self-reported patient-centered care for community-dwelling older persons (*N* = 117).

	**Patient-centered**		**Not patient-centered**	
**In general, during your appointments with health care professional do they:**	**Yes and I wanted this**	**No, but I didn't want this**	**Yes, but I didn't want this**	**No, but I wanted this**
Listen to what you have to say?	91 (78%)	3 (3%)	7 (6%)	15 (13%)
Encourage you to ask them questions?	71 (61%)	9 (8%)	7 (7%)	29 (25%)
Give you enough time to explain your health concerns?	87 (75%)	1 (1%)	6 (5%)	22 (19%)
Do whatever they can to address your health concerns?	93 (82%)	2 (2%)	5 (4%)	13 (11%)
Explain things in a way you can understand?	94 (81%)	2 (2%)	5 (4%)	15 (13%)
Mainly speak to you, rather than the person/s accompanying you (e.g., Family member or friend)	86 (78%)	4 (4%)	6 (5%)	15 (14%)
Speak to you with respect	108 (94%)	0 (0%)	6 (5%)	1 (1%)
**When discussing treatments do your health care professionals:**				
Ask you about your goals or expectations of treatment?	60 (52%)	11 (10%)	2 (2%)	42 (37%)
Ask you how involved you would like to be in making decisions about treatment?	67 (58%)	10 (9%)	5 (4%)	33 (29%)
Give you sufficient information about each treatment option?	87 (75%)	2 (2%)	6 (5%)	21 (18%)
Help you weigh up the pros and cons of different treatment options?	83 (72%)	4 (3%)	5 (4%)	24 (21%)

*N varied from 111 to 116 due to missing data*.

## Discussion

This study sought to explore of the perceptions of older community dwelling adults regarding whether they had received patient-centered care from their care providers. The majority of older adults perceived that they had received care in alignment with their preferences across a range of care aspects. Nevertheless, the findings do highlight that a substantial proportion of older people perceive that some aspects of care are not delivered in alignment with their preferences. Only, 42% of participants reported receiving all 11 aspects of care in alignment with their wishes. This finding aligns with previous research examining patient-centered care aspects in oncology (13).

The only care aspect where patients reported more than 90% patient-centered care was for healthcare professionals speaking to the patient with respect. However, even for this widely endorsed care item, six patients (5%) indicated they received this but did not want it. Although on face value this seems counter intuitive, it may be that a more paternalistic healthcare approach is preferred by some patients. This may also be the case for the small proportion of participants who indicated they did not want to be listened to. These patients may also require assistance in increasing their health literacy to inform their expectations surrounding patient-doctor interactions. These findings highlight the need for health professional to elicit older patients' care preferences to enable patients to be engaged in their health management at a level that the patient wants.

Being asked about treatment goals and involvement in treatment decision-making were the aspects of care least frequently perceived as being patient-centered, with 37 and 29% (respectively) of community-dwelling older adults indicating that they did not receive these aspects, despite wanting them. This finding aligns with previous research suggesting a lack of patient engagement in treatment decision-making, particularly for older adults (9, 23). Healthcare providers have reported a perception that older adults prefer to defer their decision-making to their provider (24), which has been previously suggested in literature that could now be considered outdated (25). However, the current study indicates a strong preference for being asked about involvement in treatment decision-making by older adults, with 87% indicating they wanted this. There is a clear need to increase older patient's involvement in decision-making by healthcare professionals.

A quarter of the older adults in this study reported not being encouraged to ask questions. Encouraging patients to ask questions enables the provider to gain an understanding of the patients' health literacy and increase the probability for information retention (26). It may also reduce the likelihood of medical errors, such as medication non-adherence, and the need for follow-up calls or consultations. A previous systematic review demonstrated that interventions to increase questions by patients had small benefits and indicated a need for more extensive and targeted training for providers in addressing patient concerns (27). The findings of this study support the need for providers to consider their role in patients' asking questions and that older adults wish to have greater encouragement to do so.

## Limitations

The findings of this study should be considered in light of its limitations. The small sample size may impact on the representativeness of the study findings. While the survey was examined for general acceptability, it has not undergone rigorous psychometrics testing to determine the validity and reliability of the administered items. Further, no data was collected regarding the nature of the healthcare appointments older adults were attending, so it is not clear from the current study the healthcare settings for which these findings are applicable. However, the general nature of study questions were intended to provide an overall picture of healthcare interactions for community-dwelling older adults rather than targeting a specific interaction. It is also important to consider the participants' risk of bias in the current study. In the healthcare system, patients may feel an unequal power balance in the patient-provider relationship and, in turn, report higher levels of satisfaction with care. Understanding the health literacy of patients in this study could have further informed whether patients felt sufficiently knowledgeable on the health care they are entitled to receive.

## Implications and Future Research

The findings from this study indicate gaps in patient-centered care for community-dwelling older adults. The majority of the gap was attributed to patients not receiving an aspect of care that they wanted. This may indicate that healthcare professionals are adopting a paternalistic approach for some aspects of care. Future research should examine strategies that increase healthcare provider delivery of patient-centered care, particularly in aspects such as treatment decision making and goal setting. Research should also investigate the health literacy of patients reporting patient-centered care to inform strategies to empower older people to stipulate their expectations regarding patient-centered care. Consideration of population strategies to address healthcare culture, health literacy and health behavior represent a significant area for future consideration in working toward a true patient-centered care model in healthcare.

## Conclusion

Older people are the most frequent users of the health care system and experience a high burden of disease. This study indicated that important aspects of care are not being provided with a patient-centered approach by health care professionals. There is a need to improve healthcare providers' elicitation of older patients' preferences for care to ensure patients are able to be proactive in managing their health and hence increase the likelihood of their healthcare needs being met.

## Data Availability Statement

The raw data supporting the conclusions of this article will be made available by the authors, without undue reservation.

## Ethics Statement

Ethics approval was granted by the University of Newcastle Human Research Ethics Committee (H-2017-0356). The patients/participants provided their written informed consent to participate in this study.

## Author Contributions

RS-F designed the study. BH undertook the study. MC completed the statistical analysis. All authors contributed to manuscript writing. All authors contributed to the article and approved the submitted version.

## Funding

This work was supported by a National Health and Medical Research Council Dementia Research Team Grant (APP1095078). BH was supported by a Colin Dodds Australian Rotary Health Postdoctoral Fellowship (G1801108).

## Conflict of Interest

The authors declare that the research was conducted in the absence of any commercial or financial relationships that could be construed as a potential conflict of interest.

## Publisher's Note

All claims expressed in this article are solely those of the authors and do not necessarily represent those of their affiliated organizations, or those of the publisher, the editors and the reviewers. Any product that may be evaluated in this article, or claim that may be made by its manufacturer, is not guaranteed or endorsed by the publisher.
